# Downstream tests, treatments, and annual direct payments in older men cared for by primary care providers with high or low prostate-specific antigen screening rates using 100 percent Texas U.S. Medicare public insurance claims data: a retrospective cohort study

**DOI:** 10.1186/s12913-016-1265-1

**Published:** 2016-01-15

**Authors:** Preeti Zanwar, Yu-Li Lin, Yong-Fang Kuo, James S. Goodwin

**Affiliations:** 1 Sealy Center on Aging, University of Texas Medical Branch, Galveston, TX USA; 2 Department of Preventive Medicine and Community Health, University of Texas Medical Branch, Galveston, TX USA; 3 Department of Internal Medicine, University of Texas Medical Branch, Galveston, TX USA; 4 Present address: Department of Mathematics and Statistics, University of Houston - Downtown, Houston, TX USA

**Keywords:** Costs and cost analysis, Health services, Medicare, Health policy, Economic models, Cancer screening, Geriatrics, Prostate cancer, Prostate-specific antigen, Healthcare providers

## Abstract

**Background:**

All authorities recommend against prostate specific antigen (PSA) screening in men 75 years and older. However, some primary care physicians (PCPs) continue to have high rates of PSA, with large variation in testing. We assessed the tests, treatments, and payments for prostate cancer care in men aged 75 or older who have PCPs with high or low PSA testing rates.

**Methods:**

We performed a retrospective cohort study using the 2010 Medicare beneficiaries aged 75 or older in Texas, United States who had no prostate cancer in 2007–2009 and had an identifiable PCP. We first identified high vs. low PSA testing PCPs, and then grouped older men in the two PCP groups. We determined health care visits to any provider and to urologists in office and outpatient settings. We estimated the direct medical payments for prostate cancer care for diagnostics, treatments and visits to providers in 2010–2011 using the generalized gamma model with log link function.

**Results:**

In multilevel, multivariable analyses, 25.4 % (n = 550) of PCPs had PSA testing rates in men aged 75 or older that were significantly higher than the mean rate of all 2,169 Texas PCPs; 29.4 % (n = 638) had rates that were significantly lower. In all, 22,853 vs. 23,929 older men were cared for by PCPs with high vs. low testing rates. Older men cared for by high PSA rate PCPs were more likely to receive a PSA test (OR 3.64, 95 % CI 3.48–3.80), a biopsy (OR 1.16, 95 % CI 1.02–1.31), an ultrasound (OR 1.19, 95 % CI 1.07–1.32) or any radiation treatment (OR 1.31, 95 % CI 1.03–1.66) than men cared for by low PSA rate PCPs. Men with high PSA rate PCPs were 1.21 (95 % CI 1.05–1.39) times more likely to have such outpatient visits. The average annual adjusted Medicare payments for prostate cancer care was $25.60 higher for patients cared for by PCPs with high PSA test rates.

**Conclusions:**

Older men seeing PCPs with high rates of PSA testing undergo more testing and treatments for prostate cancer, with higher Medicare insurance payments. Future studies are needed to delineate whether men seeing PCPs with low testing rates likely received PSA tests from other providers.

**Electronic supplementary material:**

The online version of this article (doi:10.1186/s12913-016-1265-1) contains supplementary material, which is available to authorized users.

## Background

The United States (U.S.) Preventive Services Task Force [1], the American Cancer Society [2], the American College of Physicians [3], and the American Urological Association [4] recommend against the use of the prostate-specific antigen (PSA) test to detect prostate cancer in men aged 75 years and older or those with a life expectancy of less than 10 to 15 years, because the risk of harms outweighs potential benefits. Nevertheless, screening rates for PSA tests for prostate cancer detection remain high in clinical practice in older men in the U.S. [5–10]. The major harm is from over-diagnosis: detecting and treating cancers that otherwise would not have become clinically apparent in the patient’s lifetime [5].

A high level of variability in the PSA testing rates at the primary care provider (PCP) level in men aged 75 or older was recently found [5, 11]. Which PCP a man saw accounted for 27 % of the variance in whether he received PSA screening. One consequence of PSA testing is increased payments, not only for the PSA test but also for subsequent tests and treatments stimulated by an abnormal PSA level. Several groups have described the downstream trajectories after PSA testing in various settings utilizing nationally representative data [12–15]. These studies showed that more and frequent PSA testing in older men can increase the number of men who undergo prostate biopsy [13], increase their risk of trans-rectal ultrasound [15], increase their likelihood of being diagnosed with low or intermediate risk prostate cancer [12] and increase their odds of receiving prostate cancer treatments - either surgery, radiation or hormonal therapy [12, 13, 15]. The downstream reimbursement implications of prostate cancer screening in fee-for-service Medicare were estimated to be 72 % of the overall annual PSA screening expenditures of 447 million US dollars in 2009 [14].

Based on these findings, we sought to estimate the magnitude of downstream testing, treatments and Medicare public insurance payments for prostate cancer emanating from high-testing PCPs compared to low-testing PCPs. Our overall study approach was to identify PCPs in Texas, U.S., whose PSA testing rates in 2009 in men aged 75 or older were significantly higher or significantly lower than the mean rates for all Texas PCPs. We then divided our patient cohort into two groups: men seeing PCPs who were high users of PSA tests and men seeing PCPs who were low users of PSA tests. We then followed all male patients of those PCPs who were aged 75 and older to assess the amount of prostate cancer related tests and treatments they received in 2010 and 2011. In assigning PCPs to high or low PSA testing groups, only PSA testing ordered by that PCP was assessed, but in assessing prostate cancer related tests and treatments in 2010–2011, we included all tests and treatments ordered by any provider. The purpose of our study was to explore the rates of tests and treatments and Medicare payments for care in older men cared by high PSA test rate PCPs in comparison to low test rate PCPs.

## Methods

We had a data user agreement (#21332) with U.S. Centers for Medicare and Medicaid Services to use 100 % Texas Medicare data to conduct studies on the quality of cancer screening in Texas. Informed consent was not required as our study was a secondary analysis of de-identified patient data, including any procedure codes that were used. Our study was exempt from review by the Institutional Review Board of the University of Texas Medical Branch, Galveston, Texas, U.S. (#10-175, Evaluation of Cancer Screening Practices).

### Study design, setting, & data source

This was a retrospective cohort study of men aged 75 years and older in Texas, U.S. who had a PCP with a PSA test rate significantly higher or lower than the mean. In the U.S., Medicare is a federally funded public health insurance program for its citizens aged 65 years and older. We used the 100 % Medicare Claims and Beneficiary Summary Files for the state of Texas (2006–2011), Outpatient Standard Analytic files (OUTSAF), Carrier files and Medicare Provider Analysis and Review (MEDPAR) files.

### Construction of PCP groups with high or low PSA testing rate

We first selected men aged 75 or older as of 1/1/2009 who had Medicare Parts A and B with no health maintenance organization (HMO) enrollment between 2006 and 2009, to identify PCPs with high or low PSA test rates. We excluded patients with a history of prostate neoplasm in 2006–2008 as identified by the International Classification of Disease, 9^th^, Revision, Clinical Modification (ICD-9-CM) diagnostic codes: 185 (prostate cancer), V10.46 (personal history of prostate cancer), 222.2 (benign neoplasm of prostate), 233.4 (prostate carcinoma in situ) and 236.5 (neoplasm of uncertain behavior of prostate). We also excluded those who had prostatectomy in this period, identified by ICD-9-CM procedure codes 60.2-60.6 and Current Procedure Terminology (CPT) codes: 55801, 55810, 55812, 55815, 55821, 55842 and 55845. These men with an identifiable PCP in 2009 were then selected. For PCPs, we selected only those (2,169) whose patient panels included at least 20 men (87,351 men). PSA tests ordered by these PCPs in 2009 were assessed and the PSA testing rate for each PCP was estimated using a two-level generalized linear hierarchical model, adjusting for patient characteristics. Among the 2,169 PCPs, 550 (25.4 %) had a PSA testing rate significantly higher than the mean, and 638 (29.4 %) had a significantly lower rate. Among the 87,351 men, 25,280 (28.9 %) were cared for by the high PSA rate PCPs in 2009 and 26,910 (30.8 %) by the low PSA rate PCPs (Refer to Additional file 1).

### Construction of patient sample for assignment to high or low PCP group

#### Study participants

For the study cohort, men aged 75 years or older as of 1/1/2010, with Medicare Parts A and B and without HMO enrollment- between 2007 and 2011, and without history of prostate cancer in 2007–2009 were selected. Their PCPs in 2009 were identified and men who were cared for by the high PSA rate PCPs (N = 22,853) and those cared for by the low PSA rate PCPs (N = 23,929) were selected for this study (Refer to Additional file 2). Data analysis was performed in 2013–2014.

#### Patient characteristics

Patient demographic data obtained from the 2010 Beneficiary Summary File included age, sex and race/ethnicity. The number of comorbidities was assessed from the Medicare claims for physician services, outpatient and inpatient services from the Carrier File, OUTSAF and MEDPAR Files for 2009 [16-18]. Medicaid eligibility status was obtained from the 2010 Beneficiary Summary File. Area of residence was classified as metropolitan, non-metropolitan or rural as defined by the U.S. Department of Agriculture [19]. The education level at the zip code of residence was obtained from the 2011 American Community Survey estimates from the U.S. Census Bureau.

### Comparisons of tests, treatments and healthcare services among patients within high versus low PCP group

The number and proportion of men in the high or low PCP testing group who had any PSA screening test, prostate biopsy, ultrasound, imaging or prostate cancer treatment (including radiation therapy, prostatectomy or androgen deprivation therapy) during the 2-year study period were determined using MEDPAR, Carrier and OUTSAF files. The codes used to identify claims with such procedures are included in a Additional file 3. Two types of office visits were assessed: 1) visits specific for prostate cancer to any provider and 2) any visits to an urologist. Office visits were identified using CPT codes 99201–99205 and 99211–99215 from the Carrier and OUTSAF files. Visits specific for prostate cancer were defined by office visit claims with a primary diagnosis of prostate cancer (ICD-9-CM 185) in the Carrier files or in OUTSAF files. The Medicare reimbursements for prostate cancer care among beneficiaries were computed by summing the payments for PSA tests, biopsies, ultrasounds, imaging, prostate cancer treatments and Evaluation and Management (E&M) office visits specific for prostate cancer to any provider for each man across the two PCP groups.

### Statistical analysis

The Pearson Chi-Square tests were used to examine the differences in patient characteristics of those cared for by PCPs with high and low PSA testing rates. Differences between percentages in each outcome between PCPs with high and low PSA testing rates were also determined by Chi-Square tests. Logistic models adjusted for age, ethnicity, comorbidity, Medicaid eligibility, urban or rural residence and education at the zip code level were used to determine the effect of PCP type (high vs. low PSA testing rate) on each test, treatment and service type. The differences in annual Medicare payments for tests, treatments and E&M services were determined using the Wilcoxon rank-sum test.

### Modeling annual Medicare payments for patients

The differences in annual prostate specific payments and total Medicare payments were evaluated for patients by PCP group using the Wilcoxon rank-sum test. A generalized gamma model with log link function was used [20–22], adjusted for the aforementioned patient characteristics to estimate the mean annual prostate specific payments and the mean annual total payments for patients in the two PCP groups. The PCP was included as a random effect variable in our model. All patient characteristics were included in our model as categorical variables. We included twenty-nine comorbidities from year 2009 as dichotomous (yes/no) variables. A two-part model was used to estimate prostate specific payments because 33 % of the study subjects had no payments. In part I, a logistic regression model was utilized to determine the probability of having any payments. In part II, the mean annual payment per patient for those who incurred any payment, was modeled using a generalized gamma regression. The estimates from the two-part model and the generalized gamma model were similar (Table 3). SAS statistical software (Version 9.3; SAS Institute, Inc., Cary, NC) was used for all analyses. Two-sided tests of statistical significance were employed and p-values less than 0.05 were considered statistically significant.

## Results

Among the 2,169 PCPs with at least 20 men aged 75 or older in their patient panels in 2009, 550 (25.4 %) had PSA test rates significantly above the mean and 638 (29.4 %) had PSA test rates significantly below the mean. The mean testing rate was 33.5 %, adjusted for patient characteristics, including age, race and ethnicity, Medicaid eligibility, rural–urban residence, education at the zip code level and comorbidity. Patients who were 75 or older in 2010 were selected to compare the differences in prostate cancer related tests and treatments between the high versus low PSA test rate PCP groups.

Table 1 presents the characteristics of the patients in these two groups. A higher percentage of patients cared for by PCPs with high PSA testing rates lived in metropolitan areas and had high education levels. Table 1 presents the percent of men in each group who underwent PSA testing in either 2010 or 2011, and the percent of men with any PSA test ordered by the man’s PCP or by another provider. Of the men with low-testing PCPs, 33.3 % had PSA tests ordered by those PCPs, compared to 71.8 % of men with high-testing PCPs. When considering PSA screening ordered by a provider other than a patient’s PCP, 32.8 % of men with low testing PCPs and 25.4 % with high testing PCPs underwent PSA screening.

**Table 1:** Patient characteristics overall and by PCPs with high or low PSA ordering rates

		N (%)		
Characteristics	Overall	High rank PCP	Low rank PCP	*p*-value
Overall	46782 (100)	22853 (100)	23929 (100)	
Any PSA testing	32697 (69.9)	18889 (82.7)	13808 (57.7)	<0.001
Any PSA testing ordered by patient’s PCP	24378 (52.1)	16403 (71.8)	7975 (33.3)	<0.001
Any PSA testing ordered by provider other than patient’s PCP	13649 (29.2)	5810 (25.4)	7839 (32.8)	<0.001
Age Years)				
75–79	22024 (47.1)	10900 (47.7)	11124 (46.5)	0.008
80–84	14958 (32.0)	7287 (31.9)	7671 (32.1)	
≥ 85	9800 (20.9)	4666 (20.4)	5134 (21.5)	
Ethnicity				
Non-Hispanic White	38169 (81.6)	18673 (81.7)	19496 (81.5)	<0.001
Black	1346 (2.9)	586 (2.6)	760 (3.2)	
Hispanic	6614 (14.1)	3209 (14.0)	3405 (14.2)	
Others	653 (1.4)	385 (1.7)	268 (1.1)	
Elixhauser Comorbidity				
0	6110 (13.1)	2896 (12.7)	3214 (13.4)	0.027
1	12846 (27.5)	6230 (27.3)	6616 (27.6)	
2	11988 (25.6)	5889 (25.8)	6099 (25.5)	
3	7098 (15.2)	3559 (15.6)	3539 (14.8)	
≥ 4	8740 (18.7)	4279 (18.7)	4461 (18.6)	
Medicaid Eligible				
Yes	4813 (10.3)	2328 (10.2)	2485 (10.4)	0.481
No	41969 (89.7)	20525 (89.8)	21444 (89.6)	
Urban/Rural				
Metro	34536 (73.8)	17847 (78.1)	16689 (69.7)	<0.001^*^
Non-Metro	11279 (24.1)	4730 (20.7)	6549 (27.4)	
Rural	960 (2.1)	272 (1.2)	688 (2.9)	
Unknown	7 (0.0)	4 (0.0)	3 (0.0)	
Percent of persons in zip code 25 or older with high school education or higher				
Q1: ≤ 74 %	11859 (25.3)	5514 (24.1)	6345 (26.5)	<0.001^*^
Q2: 74–83 %	11503 (24.6)	5009 (21.9)	6494 (27.1)	
Q3: 83–90 %	11815 (25.3)	5829 (25.5)	5986 (25.0)	
Q4: 90–100 %	11529 (24.6)	6485 (28.4)	5044 (21.1)	
Unknown	76 (0.2)	16 (0.1)	60 (0.3)	

*PCP* primary care provider, *PSA* prostate specific antigen

*Q1* quartile 1, *Q2* quartile 2, *Q3* quartile 3, *Q4* quartile 4

*p*-value was calculated using chi-square test between high- and low-PSA-rate PCP groups

^*^The unknown was excluded for chi-square test

Table 2 presents the number, rate and odds of prostate cancer related tests, treatments received and outpatient visits by the men cared for by PCPs with either high or low PSA testing rates. These tests, treatments and visits were not necessarily performed by the men’s PCPs. Compared to the men with low testing PCPs, men with high testing PCPs were more likely to be diagnosed with prostate cancer as more of them had an office visit with prostate cancer as the primary diagnosis (1.8 % vs. 1.5 %, p = 0.017). The multivariable model adjusted for patient characteristics shows that men with high testing PCPs were 21 % more likely to have such outpatient visits. Men with high testing PCPs, in comparison to low testing PCPs, had higher rates of receiving PSA tests (82.7 % vs. 57.7 %, p < 0.001), prostate biopsies (2.4 % vs. 2.1 %, p = 0.022), ultrasound (3.4 % vs. 2.9 %, p = 0.002) and radiation treatment for prostate cancer (0.7 % vs. 0.5 %, p = 0.013). The multivariable model adjusted for patient characteristics shows that men cared for by high PSA rate PCPs were more likely to receive a PSA test (OR = 3.64, 95 % CI 3.48-3.80), a biopsy (OR = 1.16, 95 % CI 1.02–1.31), an ultrasound (OR = 1.19, 95 % CI 1.07–1.32) and any radiation treatment (OR = 1.31, 95 % CI 1.03–1.66) than men cared for by low PSA rate PCPs. The proportion of patients without any prostate cancer associated utilization was significantly lower for men cared for by high PSA rate PCPs compared to those cared for by low PSA rate PCPs (OR = 0.27, 95 % CI: 0.26-0.29).

**Table 2:** Outcomes for prostate cancer care in patients by PCPs with a high or low PSA ordering rate

Outcomes Test/Treatment/Service type	N (%)		Odds Ratio (95 % CI), High vs. Low
	High (N = 22853)	Low (N = 23929)	
Diagnostic Test			
PSA test	18889 (82.7)	13808 (57.7)	3.64 (3.48, 3.80)
Biopsy	546 (2.4)	497 (2.1)	1.16 (1.02, 1.31)
Ultrasound	774 (3.4)	688 (2.9)	1.19 (1.07, 1.32)
Imaging	196 (0.9)	174 (0.7)	1.18 (0.96, 1.45)
Treatment			
Any treatment for prostate cancer	270 (1.2)	258 (1.1)	1.10 (0.93, 1.31)
Radiation	159 (0.7)	124 (0.5)	1.31 (1.03, 1.66)
Radical prostatectomy	18 (0.1)	30 (0.1)	0.67 (0.37, 1.21)
Androgen deprivation therapy	166 (0.7)	162 (0.7)	1.11 (0.89, 1.38)
Health Services			
Outpatient office visit for prostate cancer	413 (1.8)	365 (1.5)	1.21 (1.05, 1.39)
Outpatient office visit to urologists	8087 (35.4)	8434 (35.2)	0.98 (0.94, 1.02)
Without any prostate cancer associated utilization listed above	3915 (17.1)	10046 (42.0)	0.27 (0.26, 0.29)

The average adjusted rate of ordering PSA test in 550 high test rate PCPs in 2009 was 63.0 ± 10.4. The average adjusted rate of ordering PSA test in 638 low test rate PCPs in 2009 was 7.6 ± 5.2. The odds ratio was estimated by logistic models adjusted for age, ethnicity, comorbidity, Medicaid eligibility, urban or rural residence and education at the zip code level. 80 (0.17 %) men with unknown urban/rural residence or education information were excluded. Prostate cancer associated utilization did not include outpatient office visits to urologists as the reason for the visit was not limited to prostate cancer

*CI* confidence interval, *PCP* primary care provider, *PSA* prostate specific antigen

Table 3 summarizes the annual Medicare payments in patients for prostate cancer related tests, treatments and health services by high or low testing PCPs. Compared to the low-testing PCPs, the Medicare payments for men with high testing PCPs were significantly higher in PSA tests, biopsies, ultrasounds, radiation and E&M services for prostate cancer. However, the payment made for radical prostatectomy and androgen deprivation therapy did not differ between these two groups. According to the adjusted model, the estimated annual prostate cancer associated Medicare payment in men who saw PCPs with a high PSA test rate was $80.63 (95 % CI 78.13–83.22), compared to $55.01 (95 % CI 53.34–56.73) for men who saw PCPs with a low rate of testing.

**Table 3:** Medicare payments for prostate cancer care in patients by PCPs with high or low PSA ordering rates

Medicare payment	Annual payment, mean ± STD (median, Q1-Q3)		Difference (High-Low)	p-value
	High (N = 22853)	Low (N = 23929)		
PSA tests	21.04 ± 18.35 (13.18, 12.95–26.13)	13.51 ± 17.95 (12.95, 0.00–26.13)	7.53	<0.001
Biopsies	3.05 ± 23.85 (0.00, 0.00–0.00)	2.86 ± 24.30 (0.00, 0.00–0.00)	0.19	0.022
Ultrasounds	1.76 ± 10.22 (0.00, 0.00–0.00)	1.49 ± 9.53 (0.00, 0.00–0.00)	0.27	0.001
Imaging	1.29 ± 17.29 (0.00, 0.00–0.00)	1.00 ± 14.59 (0.00, 0.00–0.00)	0.29	0.109
Radiation	52.87 ± 715.37 (0.00, 0.00–0.00)	35.78 ± 576.59 (0.00, 0.00–0.00)	17.09	0.011
Radical prostatectomy	2.39 ± 139.61 (0.00, 0.00–0.00)	1.60 ± 76.29 (0.00, 0.00–0.00)	0.79	0.116
Androgen deprivation therapy	5.49 ± 77.92 (0.00, 0.00–0.00)	5.03 ± 74.10 (0.00, 0.00–0.00)	0.46	0.597
E&M services for prostate cancer	2.83 ± 26.24 (0.00, 0.00–0.00)	2.25 ± 25.25 (0.00, 0.00–0.00)	0.58	0.026
E&M services from urologists	35.39 ± 70.54 (0.00, 0.00–52.84)	34.78 ± 71.51 (0.00, 0.00–52.28)	0.61	0.353
Prostate cancer associated payment	90.72 ± 802.43 (13.18, 12.95–26.13)	63.51 ± 642.59 (12.95, 0.00–26.13)	27.21	<0.001
Overall Medicare payment	8974.60 ± 14138.80 (3696.48, 1577.69–10282.41)	8983.52 ± 14040.08 (3702.07, 1526.73–10360.21)	−8.92	0.249
	Adjusted annual payment - GLM model (95 % CI)			
Prostate cancer associated payment	80.63 (78.13–83.22)	55.01 (53.34–56.73)	25.62	
Overall Medicare payment	8358.94 (8233.38–8486.42)	8377.71 (8254.63–8502.64)	−18.77	

*PSA* prostate specific antigen, *E&M* evaluation and management, *PCP* primary care provider, *PSA* prostate specific antigen, *Q1* quartile 1, *Q3* quartile 3, *GLM* generalized linear model with log link function

*p*-values were calculated by Wilcoxon test. Medicare payments are in 2011 U.S. dollars. The prostate cancer associated payment was also analyzed by a two-part model which generated similar estimates (the difference between patients with high and low PSA rate PCPs was $25.32)

## Discussion

In this study, we compare the frequency of prostate cancer specific care and associated costs that are incurred in older men cared for by high versus low PSA test rate PCP groups. For every 10,000 men cared for by high PSA test rate PCPs, an additional 2,495 men got PSA screened, an additional 31 men underwent prostate biopsy, an additional 51 received ultrasound, an additional 18 received radiation treatment and an additional 28 outpatient visits were made for prostate cancer related concerns to any provider. Extrapolating these findings to the 448,530 men [23] in Texas, U.S. aged 75 years and over and multiplying them by the proportion of men cared for by high PSA test rate PCPs (28.9 %), an estimated 3 million U.S. dollars can be saved annually in prostate specific health care expenses if older men in Texas with high PSA test rate PCPs saw PCPs with low PSA test rates.

After accounting for patient characteristics, the adjusted annual prostate specific payment in the high PSA test PCP group was $25.62 higher than in the low test group, an approximate 47 % increase. The overall Medicare payment per beneficiary did not differ significantly between these two PCP groups (their CIs overlapped). The 47 % increase in downstream testing roughly parallels the 43 % increase in men receiving PSA tests in those cared for by high vs. low testing PCPs (82.7 % vs 57.7 %, Table 2). Men cared for by high testing PCPs were more likely to be diagnosed with prostate cancer. However, the magnitude is small (1.8 % vs. 1.5 %, Table 2). This might contribute to the small difference in downstream diagnostic tests and treatments between men in these two groups.

Our estimates of annual prostate cancer associated payments are likely underestimates of the total payments for prostate cancer care in older men. Our study estimated the direct medical costs of downstream prostate cancer care from the U.S. Medicare’s perspective. Indirect costs of care due to productivity loss from the patient’s and caregivers’ time, and travel costs for patients and their families could not be measured using the claims data. Such indirect costs are likely to be substantially more than direct costs alone. In addition, Medicare reimbursements do not reflect payments by other payers, out-of-pocket patient or family expenses or co-payments.

There are several other limitations to our study. First, we excluded men with any evidence of prostate cancer diagnosis or treatment in the prior 3 years. However, given the high prostate cancer survival rate, our study cohort might include some patients who have received and completed prostate cancer treatment before this period. When selecting subjects aged 75 or older with a 10-year continuous enrollment for the look back period, we found almost 70 % of prostate cancer patients could be captured using data from a 3-year look back period. Second, the interpretation of the analysis is based on the assumption that PSA testing was used for screening rather than diagnostic purposes. While patients with prior ICD codes specific for prostate diseases were excluded, some PSA testing may still have been used for evaluating symptoms, such as hematuria and urinary obstruction, rather than screening. Third, patient preferences influence the receipt of PSA tests, particularly in older men [24–26]. It is possible that patients with similar preferences aggregate with specific PCPs. Fourth, the cohort of patients and physicians in this study was limited to Texas, U.S. Texas is a large state in the U.S. with recognizable geographic variations in health service utilization and health care costs [27–29], and higher PSA screening rates have been reported for southern states [29]. Therefore, the study results may not be generalizable to PCPs of older men in other states. The study results may not be applicable to younger men or to those outside the Medicare fee-for-service reimbursement system, such as those in the Veteran Affairs or those in HMOs in the U.S. The strengths of this study include use of 100 % Medicare claims data for Texas which allowed for a representative large sample of all Texas Medicare enrollees.

## Conclusions

We found substantial variation in the frequency of prostate cancer specific tests, treatments, services, and associated costs in men with PCPs with high versus low PSA testing rates. Future studies are needed to explore the drivers of differences between the high versus low PSA testing PCPs and their dynamics with urologists and other providers in the delivery of efficient cancer care [30], and to examine the effect of payment reform in balancing the perspectives of various actors in health decision making in the light of the current U.S. Patient Protection and Affordable Care Act [31, 32].

## Additional files

Additional file 1: **Flowchart for construction of primary care provider sample with high versus low prostate specific antigen test ordering rate, 100% Texas U.S. Medicare public insurance claims data.** (DOC 31 kb)

Additional file 2: **Flowchart for construction of patient sample for assignment to primary care providers with high versus low prostate specific antigen test ordering rate, 100% Texas U.S. Medicare public insurance claims data.** (DOC 31 kb)

Additional file 3: **Summary of CPT, HCPCS, and ICD-9-CM codes used in our study, 100% Texas U.S. Medicare public insurance claims data.** (DOC 34 kb)

## Abbreviations

PSA: Prostate specific antigen
PCP: Primary care provider
U.S.: United States
HMO: Health maintenance organization
ICD-9-CM: International classification of disease, 9^th^, revision, clinical modification
CPT: Current procedure terminology
OUTSAF: Outpatient standard analytic file
MEDPAR: Medicare provider analysis and review file
HCPCS: Healthcare common procedure coding system
E&M: Evaluation & management
OR: Odds ratio
