# Supplementary material for: Downstream tests, treatments, and annual direct payments in older men cared for by primary care providers with high or low prostate-specific antigen screening rates using 100 percent Texas U.S. Medicare public insurance claims data: a retrospective cohort study
Source: BMC Health Serv Res. 2016 Jan 15;16:17. doi: 10.1186/s12913-016-1265-1 (PMC4715293; doi:10.1186/s12913-016-1265-1)
Supplement: Supplementary file 1 — Flowchart for construction of primary care provider sample with high versus low prostate specific antigen test ordering rate, 100% Texas U.S. Medicare public insurance claims data. (DOC 31 kb) [file 12913_2016_1265_MOESM1_ESM.doc]

Additional File 1 – Flowchart demonstrating the cohort selection for identifying PCPs who had high or low PSA ordering rate in 2009.

Cohort selection

|  | N | % of the last step |
| --- | --- | --- |
| 75+ Male Texas residents in 2009 | 411292 |  |
| ↓ |  |  |
| Complete enrollment in 2006-2009 | 270912 | 65.9 |
| ↓ |  |  |
| Exclude those with prostate cancer history in 2006-2008 | 215841 | 79.7 |
| ↓ |  |  |
| Select those with a PCP in 2009 (at least 2 visits) | 122560 | 56.8 |
| ↓ |  |  |
| Keep those men whose PCPs have 20 or more patients in the cohort | 87351  (2169 PCPs) | 71.3 |

Figure A1. Cohort selection for identifying PCPs who had high or low PSA ordering rate in 2009. Among the 2169 PCPs, 550 (25.4%) had a significant higher PSA ordering rate in 2009 and 638 (29.4%) had a significant lower rate. The mean rate is 33.5% adjusted for patient characteristics, including age, race/ethnicity, Medicaid eligibility, rural-urban residence, education at the zip code level and comorbidity.
