# Supplementary material for: Downstream tests, treatments, and annual direct payments in older men cared for by primary care providers with high or low prostate-specific antigen screening rates using 100 percent Texas U.S. Medicare public insurance claims data: a retrospective cohort study
Source: BMC Health Serv Res. 2016 Jan 15;16:17. doi: 10.1186/s12913-016-1265-1 (PMC4715293; doi:10.1186/s12913-016-1265-1)
Supplement: Supplementary file 2 — Flowchart for construction of patient sample for assignment to primary care providers with high versus low prostate specific antigen test ordering rate, 100% Texas U.S. Medicare public insurance claims data. (DOC 31 kb) [file 12913_2016_1265_MOESM2_ESM.doc]

Additional File 2 – Flowchart demonstrating the study cohort selection consisted of men aged 75 or older in 2010 cared for by the high or low PSA ordering rate PCPs.

Cohort selection

|  | N | % of the last step |
| --- | --- | --- |
| 75+ Male Texas residents in 2010 | 424919 |  |
| ↓ |  |  |
| Complete enrollment in 2007-2011, alive thru 12/31/2011 | 242767 | 57.1 |
| ↓ |  |  |
| Exclude those with prostate cancer history in 2007-2009 | 194084 | 79.9 |
| ↓ |  |  |
| Select those with a PCP in 2009 | 110518 | 56.9 |
| ↓ |  |  |
| Select those whose PCP had a significant higher or lower PSA ordering rate in 2009 | 46782  (1188 PCPs) | 42.3 |

Figure A2. Study cohort selection consisted of men aged 75 or older in 2010 cared for by the high or low PSA ordering rate PCPs.
