# Supplementary material for: Downstream tests, treatments, and annual direct payments in older men cared for by primary care providers with high or low prostate-specific antigen screening rates using 100 percent Texas U.S. Medicare public insurance claims data: a retrospective cohort study
Source: BMC Health Serv Res. 2016 Jan 15;16:17. doi: 10.1186/s12913-016-1265-1 (PMC4715293; doi:10.1186/s12913-016-1265-1)
Supplement: Supplementary file 3 — Summary of CPT, HCPCS, and ICD-9-CM codes used in our study, 100% Texas U.S. Medicare public insurance claims data. (DOC 34 kb) [file 12913_2016_1265_MOESM3_ESM.doc]

Additional File 3 – Summary of CPT, HCPCS and ICD-9-CM codes used in this study.

| Test/Treatment/Service | CPT code | HCPCS code | ICD-9-CM code |
| --- | --- | --- | --- |
| Prostate-specific antigen test | 84152-84154 | G0103 |  |
| Biopsy | 10021^a^, 10022^a^, 55700-55706, 76942^‡^, 88172^‡^, 88173^‡^ | G0416-G0419 | 60.11, 60.12 |
| Ultrasound | 76872, 76873 |  |  |
| Imaging | 3269F, 72191-72197^‡^, 76977^‡^, 78300-78320^‡^, 78350^‡^, 78351^‡^, 78399^‡^, 77002^‡^, 77012^‡^, 77021^‡^ |  | 87.92^‡^, 88.95^‡^ |
| Radiation therapy | Followed SEER recommendation ([http://healthservices.cancer.gov/seermedicare/considerations/procedure_codes.html Accessed](http://healthservices.cancer.gov/seermedicare/considerations/procedure_codes.html Accessed ) April 20 2015). Primary diagnosis must be prostate cancer. |  |  |
| Radical prostatectomy | 55801-55845, 55866 |  | 60.5 |
| Androgen deprivation therapy | 4164F, 54520, 54522, 54530, 54535, 54690 | J1950, J3315, J9202, J9217, J9218, J9219 | 62.41, 62.42 |
| Evaluation and management services | 99201-99205, 99211-99215, 99241-99245, 99251-99255, 99381-99387, 99391-99397, 99401-99404, 99411-99412, 99420-99429, 99441-99444, 99499-99456, 99304-99306, 99334-99337, 99339-99340, 99341-99345, 99347-99350, 99354-99357, 99358-99359, 99324-99328, 99334-99337, 99339-99340, 99221-99223, 99231-99233, 99238-99239, 99217-99220, 99234-99236, 99281-99288, 99291-99292, 99366-99368, 99363-99364, 99374-99380 | G1028, G0151-G0155, G0179-G0182, G0337, G0378-G0384, G0402, G0425-G0427, G0912, G9001-G9011 |  |
| Office or other outpatient services | 99201-99205, 99211-99215 |  |  |

CPT, Current Procedural Terminology.

HCPCS, Healthcare Common Procedure Coding System.

ICD-9-CM, International Classification of Diseases, Ninth Revision, Clinical Modification.

Biopsies includes transperineal stereotactic template guided, needle or punch, single or multiple.

Imaging includes bone scans, pelvic CT scans, pelvic MRI, prostate MRI and prostate radiography.

Androgen deprivation therapy includes orchiectomy and GnRH agonists – gosrelin acetate implant, leuprolide acetate, leuprolide acetate implant and triptorelin pamoate.

^a^Primary diagnosis must be prostate cancer (ICD-9-CM 185).
